# Of adding oranges and apples: how non-abstract representations may foster abstract numerical cognition

**DOI:** 10.3389/fnhum.2013.00903

**Published:** 2013-12-24

**Authors:** Andrea Bender, Sieghard Beller

**Affiliations:** Department of Psychosocial Science, University of BergenBergen, Norway

**Keywords:** numerical cognition, number systems, culture, distributed cognition, abstract mathematical thinking

## Introduction

The realm of numbers constitutes just one of many fields of mathematical cognition, but arguably a pivotal one. It is also among those core domains of knowledge that—while being prepared for unfolding in the human species (Feigenson et al., 2004; Hyde, 2011)—nonetheless requires cultural mediation to unfold to its full potential: Not only is the availability of a conventionalized counting sequence essential for accurate counting and calculating (Gordon, 2004; Pica et al., 2004; Frank et al., 2008; Spaepen et al., 2011), acquiring a counting sequence in the first place is also crucial in more fundamental ways: for grasping the concept of precise quantities, for comprehending the ordinal and cardinal nature of numbers, or for learning the algorithms of basic arithmetics that then pave the way for higher levels of mathematics.

Learning to count also promotes acquaintance with some of the more general principles that characterize mathematics such as abstractness. In fact, one of the first principles to be learned in this process is that numbers are abstract—all kinds of entities can be counted with the same number words (Gelman and Gallistel, 1978; but see also Cohen Kadosh and Walsh, 2009). But not all counting sequences seem to reflect this principle. A substantial number of Oceanic languages, for instance, have counting sequences whose usage is restricted to specific objects, while other objects are counted otherwise (Bender and Beller, 2006a,b).

This pattern of counting different things differently seems to directly contradict the abstractness principle and has thus been taken as an earlier stage in the evolution of numerical thinking (e.g., Klix, 1993). While the latter assumption was refuted elsewhere (Beller and Bender, 2008), the question remains open of how (if at all) such apparently non-abstract counting sequences may foster abstract numerical cognition. Here, we defend the position that the Oceanic counting sequences are not only *compatible* with an abstract understanding of numbers, but may even *promote* such an understanding. To this end, we propose to conceive of these sequences as the verbal components of the mathematical code, which provide the symbols that people use to represent and manipulate abstract mathematical concepts. Analyzing how the specific properties of these symbol systems affect the processing of numerical information will help us to understand better how abstract mathematical thinking emerges.

## Counting sequences and their cognitive implications

In general, each counting sequence consists of a limited set of symbols for basic numbers and (optionally) some composition rules for representing larger numbers. These symbols and composition rules constitute a distinct numeration system, the properties of which may differ substantially across languages (Chrisomalis, 2004; Bender and Beller, 2012; Widom and Schlimm, 2012).

The system's internal structure, for instance, depends on its dimensionality (Zhang and Norman, 1995). One-dimensional systems are unstructured; they either use the same symbol in a cumulative manner to indicate increasing set size (as in tallies), or employ specific symbols distinctively to indicate distinct set sizes (as with the Arabic digits from 1 through 9). In contrast, two-dimensional systems like the English number words make use of a base (in this case: “ten”), which is raised to various powers (“hundred,” “thousand,” etc.). Number words in between are composed according to the addition and multiplication principle, as in “two hundred and three.”

Counting sequences are cultural tools, whose properties may give rise to “representational effects” (Zhang and Norman, 1995), that is, they affect how numerical information is represented and processed (Nickerson, 1988; Fuson, 1990; Miller et al., 1995; Zhang and Wang, 2005; Schlimm and Neth, 2008; Domahs et al., 2010; Beller and Bender, 2011; Krajcsi and Szabo, 2012). An analysis of such representational effects will help us to illuminate the cognitive implications of specific counting systems.

## Specific counting systems in oceanic languages

In a large number of Oceanic languages, two types of verbal systems co-exist: regular systems for general counting and systems restricted to counting specific objects in a particular manner (Bender and Beller, 2006a,b). For illustration, take the Polynesian language spoken on Tonga, an island group in the Western Pacific.

Tongan employs five numeration systems: a general and four specific ones. All of them contain primary numerals for the numbers 1 through 10 and for the powers of the base up to 10^5^ (Bender and Beller, 2007). The specific systems deviate from each other and from the general system in three ways: They make use of diverging counting units; they employ distinct lexemes for some of the powers; and they are applied to only one kind of object each. Accordingly, sugar-cane is counted in pairs, whereas coconuts, pieces of yam for planting, and fish are counted in pairs when few, but in scores when numerous.

It was especially the object-specificity of counting that arrested researchers' interest early-on and nurtured the assumption that speakers of languages like Tongan may lack an abstract concept of number (Klix, 1993). However, viewing this feature in the context of the other two peculiarities allows for a more accurate assessment. It reveals that number word composition in the specific systems remains highly systematic. In fact, the rules for composing number words in the general system require only marginal modifications (namely acknowledgement of the counting unit and the specific power numerals) to generate number words in the specific systems. This structural alignment, together with the older age of the general system, also suggests that the specific systems were deliberately developed out of the general one (Bender and Beller, 2006a,b, 2007).

While the structure of the general counting sequence was retained, the counting unit to which its constituents referred (and hence the value of the counted set) was increased. This transformation of values follows the same principle that is inherently instantiated in two-dimensional systems, namely the multiplication principle for composing larger number words. In these systems, the base and its powers are counted as if they were objects: “three hundreds” is similar to “three baskets.” Specific systems carry this abstraction one step further by implicating that the “three hundreds” may refer in fact to “three hundreds of pairs or scores.”

Adopting the multiplication principle inherent in power term constructions (like “three hundreds”) for the creation of specific counting systems is far from being trivial. It requires a sophisticated understanding of counting insofar, as number words are used now to count not just objects, but other numbers and even abstract counting units. To this end, countability is defined recursively, and in doing so, also paves the way for conceiving of multiplication as an algorithm for mental arithmetics.

What at first glance may look laborious—the recursive extraction of numerical values—can, thus, in fact be cognitively advantageous: It allows for more compact representations, which, in the absence of notation, not only reduces cognitive load (Beller and Bender, 2008), but also increases the speed and correctness of mental arithmetic (Lordahl et al., 1970; Bender and Beller, 2013).

## Conclusion

With our analysis we hope to have demonstrated, that the apparently non-abstract representations in Oceanic counting systems have indeed fostered abstract numerical cognition. But beyond this rehabilitation of the specific systems and their users, this “exotic” phenomenon is of more general relevance to the cognitive sciences. It also serves as an instance of the recursive process in which cultural tools and cognitive achievements advance each other and thus as an instance of the “ratchet effect” (Tomasello, 1999; and see Wiese, 2003) of culture more generally, which also highlights the importance of anthropological insights for cognitive science theorizing (Beller et al., 2012). By their mere existence and usage, cultural tools may promote cognitive advancement. Designed to serve one purpose, tools generally have more properties than only those relevant to the task at hand, and these properties may then afford new ways of usage or reasoning (see also Miller and Paredes, 1996; Coolidge and Overmann, 2012). It is this extra value of cultural tools that, in the domain of mathematical cognition, promotes abstract thinking.
